# Pattern Formation in Two-Component Monolayers of Particles with Competing Interactions

**DOI:** 10.3390/molecules28031366

**Published:** 2023-01-31

**Authors:** Alina Ciach, Andres De Virgiliis, Ariel Meyra, Marek Litniewski

**Affiliations:** 1 Institute of Physical Chemistry, Polish Academy of Sciences, 01-224 Warsaw, Poland; 2 Instituto de Física de Líquidos y Sistemas Bilógicos, Facultad de Ciencias Exactas-UNLP-CONICET, La Plata 1900, Argentina; 3 Departamento de Ciencias Basicas, Facultad de Ingeniería, Universidad Nacional de La Plata, La Plata 1900, Argentina; 4 Departamento de Ingeniería Mecánica, Facultad Regional La Plata, Universidad Tecnológica Nacional, La Plata 1900, Argentina

**Keywords:** mixture of charged particles, competing interactions, self-assembly, pattern formation, thermodynamic Casimir potential, molecular modeling

## Abstract

Competing interactions between charged inclusions in membranes of living organisms or charged nanoparticles in near-critical mixtures can lead to self-assembly into various patterns. Motivated by these systems, we developed a simple triangular lattice model for binary mixtures of oppositely charged particles with additional short-range attraction or repulsion between like or different particles, respectively. We determined the ground state for the system in contact with a reservoir of the particles for the whole chemical potentials plane, and the structure of self-assembled conglomerates for fixed numbers of particles. Stability of the low-temperature ordered patterns was verified by Monte Carlo simulations. In addition, we performed molecular dynamics simulations for a continuous model with interactions having similar features, but a larger range and lower strength than in the lattice model. Interactions with and without symmetry between different components were assumed. We investigated both the conglomerate formed in the center of a thin slit with repulsive walls, and the structure of a monolayer adsorbed at an attractive substrate. Both models give the same patterns for large chemical potentials or densities. For low densities, more patterns occur in the lattice model. Different phases coexist with dilute gas on the lattice and in the continuum, leading to different patterns in self-assembled conglomerates (‘rafts’).

## 1. Introduction

Some time ago it was observed that multicomponent biological membranes in living organisms are close to the critical point of the miscibility phase transition [1,2,3,4]. At the critical point located at the end of the coexistence line, the difference between the coexisting phases vanishes, but concentration fluctuations are correlated over macroscopic distances. When the critical point is approached from the phase with mixed components, the correlation length between the concentration fluctuations grows, and depends very sensitively on temperature.

It is well-established theoretically and experimentally [5,6,7,8,9,10] that the correlations between concentration fluctuations lead to the so-called thermodynamic Casimir interaction between objects immersed in the near-critical mixture. The range of the Casimir potential is equal to the correlation length of the concentration fluctuations, i.e., it can be tuned by changing the temperature. The Casimir interaction is attractive or repulsive between two objects preferentially adsorbing the same or different components of the near-critical mixture, respectively [6,7,8,9]. Thus, large macromolecules embedded in the biological membrane can attract or repel each other with the Casimir potential if they preferentially adsorb the same or different types of lipids, respectively [4].

In biological systems, macromolecules such as proteins are often charged, and repel or attract each other with screened electrostatic interactions when they have like or opposite charges, respectively. Thus, charged macromolecules embedded in the biological membrane interact with the sum of the electrostatic and the Casimir potentials.

The competing interactions described above are not limited to the charged proteins anchored in the biological membranes, but are rather common in soft-matter systems, where charged particles are dissolved in complex solvents. Charged nanoparticles immersed in a binary liquid mixture intermixed with salt and deposited on a substrate, and oppositely charged colloid particles with hydrophilic and hydrophobic surfaces immersed in a near-critical water-lutidine mixture are representative examples of one- and two-component systems with competing interactions [8,10,11].

When the correlation length of the concentration fluctuations in the solvent is shorter than the screening length of the electrostatic interactions, then like particles or macromolecules may attract and repel each other at short and long distances, respectively. However, the oppositely charged particles or macromolecules may repel each other at short and attract each other at long distances. Not too close to the critical point and with a relatively low density of ions in the solvent, we may expect short-range attraction and long-range repulsion (SALR) between like-charge particles, and short-range repulsion and long-range attraction between oppositely charged particles, when the magnitude of the Casimir potential is larger than the magnitude of the screened electrostatic interaction [8,9,10,11,12,13].

One-component systems with SALR (or ‘mermaid’) potentials have been extensively studied by experiment, theory, and simulations in three (3D) and two (2D) dimensions [14,15,16,17,18,19,20,21,22,23,24,25,26,27,28,29,30,31]. It was found that for increasing density of the particles, the same sequence of ordered phases appears for different shapes of the SALR potential [18,19,32]. In two dimensions, in addition to the disordered dilute and dense phases, a hexagonal arrangement of clusters, next stripes, and finally a hexagonal arrangement of bubbles are stable for increasing density [18,19,20]. Only the thickness of the aggregates depends on the shape of the SALR potential. Interestingly, the patterns obtained in 2D models agree with the patterns formed by the SALR particles adsorbed on a solid substrate [11,33]. Moreover, the adsorbed monolayer is followed by a depletion zone [33].

Binary mixtures with competing interactions have attracted much less attention so far. The structure of the disordered phase in 3D was studied by simulations and theory [34,35,36,37,38,39,40]. The phase diagram in 3D was determined theoretically and by simulations for a symmetrical mixture with equal numbers (or chemical potentials) of the particles of the first and the second component, for the interactions inspired by the interactions between the charged macromolecules anchored in the membrane [37]. It was found that the dilute gas coexisted with a dense phase with alternating bilayers of the first and the second component. The adsorption from a dilute gas phase on a surface attracting the first component turned out to be significantly different than in the one-component system. Namely, several layers of particles with quite complex patterns were adsorbed, and the amounts of the first and the second component in the adsorbed film were similar [33].

The structure in the case of asymmetry in the interaction potentials and particle sizes was studied for electrostatic interactions between the particles or colloid particles in 3D [38,41], but in this case particles, not clusters, form periodic patterns. As far as we know, two-component monolayers of particles with competing interactions having the properties described above have not yet been studied theoretically. According to the experimental observations, the miscibility critical point in membranes of living cells belongs to the two-dimensional Ising universality class [4]; therefore, the pattern formation by the membrane-bound inclusions can be investigated in two-dimensional models.

In this work, we study the general qualitative features of the self-assembly in such types of mixtures rather than any specific case. The results obtained for the SALR-type of model show that the qualitative behavior of the mixtures with competing interactions can be determined by studies of simple model systems. Based on this expectation, we develop a generic 2D model for the mixture of charged particles dissolved in the near-critical solvent that is as simple as possible. In the simplest generic model of such a mixture, the particles occupy cells of a triangular lattice. Like particles interact with the potential V(x) that is negative and positive for x=|x|=1a and x=2a, respectively, and vanishes for x>2a, where *a* is the lattice constant, whereas the interaction between the particles of the first and the second component is −V(x). The model is an extension of the model for the one-component SALR system developed and studied in refs. [19,20]. Within our new model, we determine the patterns minimizing the energy for a fixed number of particles of each component, as well as for a system in contact with a reservoir of the particles, i.e., with fixed chemical potentials.

The main question that we address here is what types of ordered patterns can be stable at low temperature *T* for given numbers of particles or for given chemical potentials. More precisely, we are interested in the ordered structures formed when the absolute value of the interaction energy per particle is significantly larger than the thermal energy $k_{B}T$, where $k_{B}$ is the Boltzmann constant. Such ordered patterns correspond to the minimum of the energy, and can be determined by the analysis of the ground state (GS) with T=0 for selected numbers of particles and interaction strengths. To discover which patterns are stable in an open system, we determine the GS of the model for the whole plane of the chemical potentials. When T>0, thermal motion leads to the formation of defects, and such imperfect structures are studied by Monte Carlo simulations. Here, we are not interested in the disordered phases with delocalized aggregates that are expected at higher *T*.

In order to verify if the lattice structure and the symmetry of the interactions have a strong influence on the results, we compare our predictions with the molecular dynamics (MD) simulation results for a continuous model with and without symmetry of the interactions. In the second case, we assume that the charge of the second component decreases by a factor *q* compared to the first component, and rescale the short-range interactions in the same way. In this case, we analyze the structure in the layers of particles self-assembled between two parallel repulsive planes separated by a distance equal to 4 particle diameters, and inside the films adsorbed at an attractive solid substrate.

In Section 2 we introduce our models. The methods are briefly described in Section 3, and in Section 4 our results are presented and discussed. Section 5 contains a summary and conclusions.

## 2. The Models

### 2.1. The Lattice Model

We consider spherical particles with equal diameters on a triangular lattice. Multiple occupancy of the lattice cells is forbidden to mimic the hard cores of the particles. For this reason, the model is not suitable for soft particles with cores that can overlap. The particle interactions are assumed to be symmetrical with respect to particle identities, i.e., the first-neighbor attraction between like particles is $-J_{1}$ and the repulsion between different species is $J_{1}$. For the second neighbors the interactions change sign, and for the third neighbors we assume $J_{2}$ and $-J_{2}$ for the same and different species, respectively. For distances r>a, where *a* is the lattice constant assumed to be equal to the particle diameter, the interaction potential between the particles separated by the distance Δx is

(1) $$V_{ij}\left(\Delta x\right)=\left\{\begin{array}{c} \;V(\Delta x)\;\mathrm{for}i=j\;\\ -V(\Delta x)\;\mathrm{for}i\neq j\; \end{array}\right.$$

with i,j=1,2 referring to the first and the second component, and

(2) $$V\left(\Delta x\right)=\left\{\begin{array}{c} -J_{1}\;\mathrm{for}|\Delta x|=1,\;(\mathrm{for}\mathrm{nearest}\mathrm{neighbors})\\ +J_{2}=JJ_{1}\;\mathrm{for}|\Delta x|=2,\;(\mathrm{for}\mathrm{third}\mathrm{neighbors})\\ 0\;\;\mathrm{otherwise}. \end{array}\right.$$

**Figure 1:**
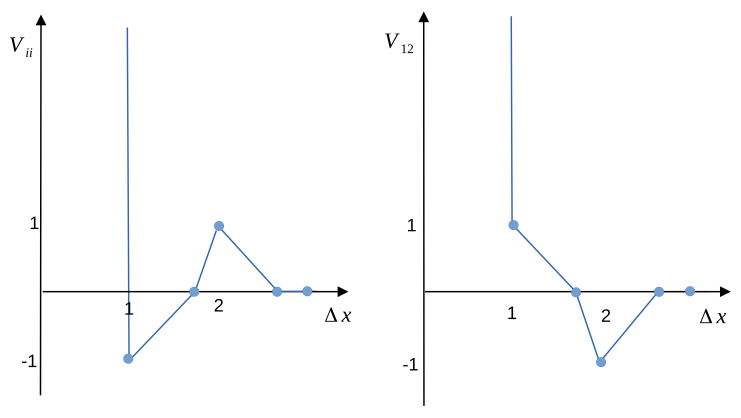
The interactions given in (1) and (2) in units of $J_{1}$ for $J=J_{2}/J_{1}=1$. The (**left**) and (**right**) panels correspond to the interaction between like and different particles, respectively. The filled circles indicate the interactions between the particles occupying the lattice cells on the triangular lattice, and the lines are to guide the eye. The vertical lines at Δx=1 represent the infinite repulsion for overlapping cores of the particles.

Here and below, the length is measured in units of *a*. We assume that the energy unit is given by the nearest-neighbor interaction $J_{1}$, and will use the dimensionless energy $V^{*}\left(\Delta x\right)=V\left(\Delta x\right)/J_{1}$, dimensionless temperature $T^{*}=k_{B}T/J_{1}$, and dimensionless chemical potentials $\mu_{i}^{*}=\mu_{i}/J_{1}$. The relevant dimensionless parameter associated with the shape of the interactions is the ratio $J=J_{2}/J_{1}$. The interaction potential between like and different particles is shown in Figure 1 for J=1.

The interaction potential (2) leads to a relatively small range of attraction between like particles, therefore when small clusters of like particles are formed, the energy decreases. The range of the repulsion is also small, therefore the clusters of like particles can be located rather close to each other with no energetic cost of the repulsion. However, nearest neighbors of different kinds lead to an increase in the energy, while the energy decreases when different particles are separated by Δx=2.

### 2.2. The Continuous Model

**Figure 2:**
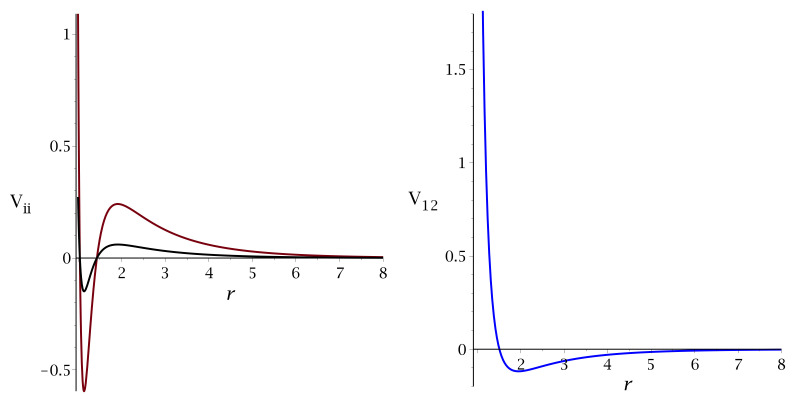
The interactions given in (3) in units of ϵ for q=1/2. The (**left**) and (**right**) panels correspond to the interaction between like and different particles, respectively. The red and black lines correspond to the first and the second component particles, respectively.

In the lattice model, the particle positions are restricted to the lattice cells, and we assume that the interactions between like particles are the same for the particles of the first and the second component. In order to examine the effect of the lattice structure, we develop here a continuous model. In addition, to determine the role of the symmetry in the interaction potentials, we assume different interactions between two particles of the first component, and between two particles of the second component. Finally, we consider a fixed number of particles. The patterns formed in this model are determined by MD simulations, therefore the hard cores are replaced by the repulsive part $1/r^{12}$ of the Lennard–Jones (LJ) interaction potential, Equation (3). In Refs. [33,37], we studied a symmetrical mixture with like particles interacting with the Lennard–Jones potential plus the screened electrostatic repulsion. Here, we assume that the interaction potentials have the following form:

(3) $$V_{ij}\left(r\right)=6\epsilon \left\{\begin{array}{c} \left(r^{-12}-r^{-6}+0.3e^{-r/2}/r\right)\;\;\mathrm{for}i=j=1\;\\ \left(r^{-12}-r^{-6}+0.3e^{-r/2}/r\right)q^{2}\;\mathrm{for}i=j=2\;\\ \left(r^{-12}+r^{-6}-0.3e^{-r/2}/r\right)q\;\mathrm{for}i\neq j,\; \end{array}\right.$$

where *r* is the interparticle distance in units of the particle diameter *a*. The potential is truncated at the cutoff r=6.75. With this potential (see Figure 2), we do not try to model any particular system, since our aim is to determine general trends in pattern formation in a binary mixture with competing interactions.

In the continuum case, the repulsion between like particles is weak but of a long range, whereas in the lattice model, strong but relatively short-range repulsion is present for J>1. We will verify if this difference in the repulsive part of the interactions can influence the self-assembled structures in the binary mixture.

## 3. Methods

### 3.1. The Lattice Model Calculations and Simulations

We separately consider the system with a fixed number of particles $N_{i}$ of the component *i*, and the open system that is in contact with the reservoir of particles with fixed chemical potentials $\mu_{1,2}$.

For fixed $N_{i}$, we limit ourselves to $N_{i}\ll A$, where *A* is the number of lattice cells. We simply calculate the energy for different patterns formed by the particles on the lattice by counting the pairs of cells separated by Δx=1 and Δx=2 that are occupied by like and different particles, and summing up the contributions to the energy.

In the open systems that are in contact with the reservoir of particles, the equilibrium state corresponds to the minimum of the thermodynamic Hamiltonian. For our model, it has the following form (in $J_{1}$ units):

(4) $$H^{*}=h^{*}A=\frac{1}{2}\sum_{x}\sum_{x^{\prime }}{\hat{\rho }}_{i}\left(x\right)V_{ij}^{*}\left(x-x^{\prime }\right){\hat{\rho }}_{j}\left(x^{\prime }\right)-\mu_{i}^{*}\sum_{x}{\hat{\rho }}_{i}\left(x\right)$$

where $\sum_{x}$ is the sum over all lattice cells, and the summation convention for repeated indexes is used. ${\hat{\rho }}_{i}\left(x\right)$ is the occupation number for *i*-th-type particles, i.e., ${\hat{\rho }}_{i}\left(x\right)=1$ or 0 if the site x is occupied by the *i*-th-type particle or not, respectively, and we require that ${\hat{\rho }}_{1}\left(x\right){\hat{\rho }}_{2}\left(x\right)=0$, i.e., two different particles cannot occupy the same lattice site.

In order to find patterns that minimize $H^{*}$ for given $\left(\mu_{1}^{*},\mu_{2}^{*}\right)$ at $T^{*}=0$, we consider several possible ordered structures and calculate $h^{*}$ for all of them. To find $h^{*}$ for ordered structures, we identify the unit cell of the periodic pattern and assume periodic boundary conditions. The equilibrium pattern corresponds to the lowest $h^{*}$ for the considered $\left(\mu_{1}^{*},\mu_{2}^{*}\right)$.

Like in most systems showing self-assembly, the role of mesoscopic fluctuations on pattern formation in thermal equilibrium of SALR potentials is very important, since they dictate the distribution and stability of the different structural motifs and phases.

In order to explore these effects, we performed Monte Carlo (MC) simulations of the model defined above (see Equations (1) and (2)) on a triangular lattice, for some specific values of the parameter $J=J_{2}/J_{1}$. The grand canonical ensemble was employed to simulate the mixture at fixed chemical potentials $\mu_{1}$, $\mu_{2}$ of the species, in such a way that representative states are to be compared with those found in the ground-state calculations. In addition, simulations at constant $N_{1},N_{2}$, i.e., in the canonical ensemble (NVT), were applied to explore the influence of the size of the assemblies in the aggregation process.

Based on the well-known equivalence between Ising and lattice gas models [42], we define at every lattice site an Ising-like variable *S* taking the values +1,−1,0 to represent the species 1 and 2, and an empty site, respectively. We only consider particle insertion and removal moves for each species. Other types of moves, like particle identity exchange (1↔2) or cluster moves, are not employed at this time.

Since the main interest of the present work is to extract the topology of the phase diagram at low temperature, we avoided applying more sophisticated simulation strategies devised to accurately determine phase boundaries, like parallel tempering or thermodynamic integration. We only adopted the standard Metropolis sampling scheme in such a manner that at every single step we tried to add (remove) a particle of the i− type with the probability

(5) $$p\left(i\right)=\left\{\begin{array}{c} \exp(u_{i}+\mu_{i})\;\;\mathrm{for}\mathrm{insertion}\\ \exp(-\left(u_{i}+\mu_{i}\right))\;\mathrm{for}\mathrm{removal} \end{array}\right.$$

where $u_{1}=-u_{2}=J_{1}\left(\Delta_{1}-3\Delta_{2}\right)$ with $\Delta_{1},\Delta_{2}$ denoting the sums of the *S* variable on the first- and third-neighbors.

We simulated triangular lattices of lateral size L=60 with periodic boundary conditions. An MC step is defined as $N=L^{2}$ attempts of inserting or removing a particle. The approach to the ground state was realized by starting every simulation at fixed values of ${\mu_{1}^{*},\mu_{2}}^{*}$ with an empty lattice and at a rather high temperature $T^{*}=5.0$ (in units of $J_{1}$). Then, through a sequence of simulations at decreasing temperature, we reached the final low temperature $T^{*}=0.2$. Depending on the values of the chemical potentials involved, each of these simulation paths comprised the first equilibration period of 10${}^{5}$–10${}^{6}$ MC steps, followed by the production period of 10${}^{6}$–10${}^{7}$ steps, where the configurations of the lattice were stored.

For the simulations in the canonical ensemble (NVT), we employed the same strategy for thermalization of the system, with the only exception that now the stochastic evolution of the system is promoted through displacement moves to empty neighbor sites. The transition probabilities associated with these moves were adapted from Equation (5) with suitable modifications [43].

### 3.2. The Continuous Model Simulations

We intend to compare patterns formed by macromolecules anchored in a membrane with patterns formed by particles inside a film adsorbed on a solid surface. To model the two cases, we consider a rectangular box with periodic boundary conditions in the x,y directions, and with two confining walls at z=0 and $z=L_{z}$.

To impose conditions supporting formation of a monolayer, we assume that the two confining walls are close to each other ($L_{z}=4$), and the sizes of the box in the horizontal directions are $L_{x}=L_{y}=150$. Both walls are repulsive for both components with the wall–particle interaction of the form

(6) $$V_{rep}\left(z\right)=\frac{\epsilon }{2{\left(z-z_{w}\right)}^{12}},$$

where $z_{w}=0$ or $z_{w}=4$.

To study the adsorption process, we considered two types of the wall at z=0. In the first case, the wall at z=0 attracts only the first component with the interaction potential

(7) $$\begin{array}{c} V_{attr}\left(z\right)=4\epsilon \left(\frac{1}{z^{12}}-\frac{1}{z^{6}}\right), \end{array}$$

and the second component is repulsed according to Equation (6) with $z_{w}=0$. In the second case, both components are attracted to the substrate according to Equation (7). For both types of the wall at z=0, the distant wall at $z=L_{z}=800$ repels the particles according to Equation (6) with $z_{w}=L_{z}$. In directions parallel to the walls, $L_{x}=L_{y}=200$.

We performed molecular dynamics simulation at constant volume [44], using the same procedure as described in detail in ref. [33,37]. To study the self-assembly in the thin slit, we assumed $N_{1}=N_{2}=200$ and q=1 or q=1/2. In the case of the adsorption on a solid substrate, we assumed $N_{1}=qN_{2}$ to satisfy the ’charge neutrality’ condition. For the adsorption at the wall attracting only the first component, we simulated the system of $N_{1}+N_{2}$ = 32,001 particles (i.e., $N_{1}=10,667$ for q=1/2). Finally, for the wall attracting both components, we considered two cases: $N_{2}=2N_{1}$ = 21,334 with q=1/2, and $N_{2}=3N_{1}$ = 24,000 with q=1/3.

The ordered patterns that we studied are formed at low temperature. Because of the asymmetry in interactions, the crystallization temperature for the second component is $q^{2}$ times lower than for the first one, and it has a strong effect on the dynamics. Because we studied low-temperature structures, and at low temperature the adsorption process is very slow, we used a trick to speed it up. At the initial stage of the simulations, the simulation box was divided into three temperature regions: $\bar{T}=0.04$ for 0<z<80, $\bar{T}=0.13$ for 80<z<320, and $\bar{T}=0.17$ for z>320. We chose $\bar{T}=k_{B}T/\epsilon$ for the dimensionless temperature for this model. The mean temperature was imposed by scaling particle velocities in the same way as in ref. [37]. At the next stage of the simulations we fixed the temperature in the whole system to a low value and kept it constant for a long time.

Each of the simulations discussed in this paper ended up with a very long stationary stage in which the pattern did not change and the potential energy fluctuated around a constant value. We expect that the obtained patterns are the equilibrium ones.

## 4. Results and Discussion

### 4.1. The Lattice Model

#### 4.1.1. The Case of Fixed Numbers of Particles

**Figure 3:**
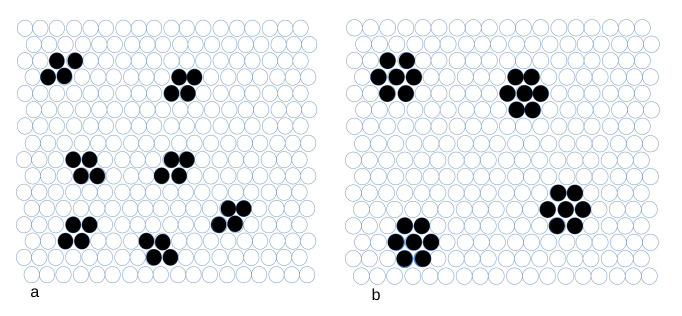
Representative configurations of 28 particles minimizing the energy for the repulsion-to-attraction ratio J>13/12 (**a**) and J<13/12 (**b**). Empty and filled circles represent empty and occupied cells of the triangular lattice. See the main text for more details.

The one-component model with the interaction potential (2) was thoroughly studied in refs. [19,20]. It was found that in the one-component system with N≪A, the energy took a minimum when the particles assembled into clusters separated by distances sufficiently large to ensure that there was no interaction between them. Such large distances between the clusters are possible only when a small number of particles is present in the system. Representative configurations minimizing the energy for 28 particles are shown in Figure 3. One can easily see that each small cluster contributes $-5J_{1}$ to the energy when the remaining clusters are at the distance larger than the range of the repulsion, and each cluster made of 7 particles contributes $\left(-12+3J\right)J_{1}$ to the system energy when the other clusters are far away. When the repulsion for Δx=2 is strong, J>13/12, then the optimal clusters consist of 4 particles, whereas for weaker repulsion, hexagonal clusters composed of 7 particles lead to the lowest energy. This is because the number of particles that can form either small or large complete clusters is (4·7)n=28n, where *n* is an integer (see Figure 3). The difference in energy of 28 particles is −35−4(−12+3J)=13−12J in $J_{1}$ units. Note that the ground state is strongly degenerated, because the clusters can occupy different cells for the same energy.

**Figure 4:**
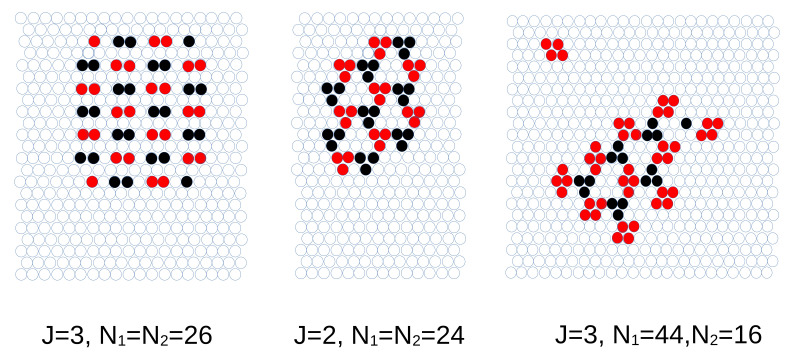
Representative patterns self-assembled for a fixed number of particles and $T^{*}\to 0$, inferred from canonical MC simulations at $T^{*}=0.2$. The numbers of particles and the ratio of the interactions between the third and the first neighbors are indicated in the figure. Empty and filled circles represent empty and occupied cells of the triangular lattice, with red and black colors referring to the first and the second component particles.

When both types of particles are present, the self-assembly changes completely, because the dispersion of small clusters in the case of $N_{i}\ll A$ is no longer energetically favored. This is because the energy decreases when particles of different species are located at the distance Δx=2. It is not obvious which patterns may lead to the lowest energy for $N_{1}=N_{2}\ll A$, therefore we performed MC simulations at $T^{*}=0.2$. The MC simulation results indicate that for J=2 and J=3 the energy takes the minimum or is very close to the minimum for the configurations shown in Figure 4. We also considered different numbers of particles and different values of *J*, and in all cases we obtained a single “porous raft”.

The particles at the boundary have fewer neighbors than the particles inside the raft, therefore it is energetically favorable to have as few particles at the boundary of the raft as possible. For structures with no rotational symmetry, the energetic cost at different boundary layers can be different, and this can lead to asymmetric shapes of the rafts.

When the number of particles inside the raft is significantly larger than the number of particles at the boundary, then the structure inside the raft should be the same as the structure of the phase coexisting with the gas (vacuum at T=0). We verified that for significantly larger $N_{i}$ the same patterns inside the raft as shown in Figure 4 (left and central panels) were obtained. Thus, the patterns shown in Figure 4 can be expected for the phases coexisting with the vacuum. The above hypothesis is verified in Section 4.1.2, where the phase coexistence between the vacuum and ordered phases is determined.

When $N_{1}\ll N_{2}\ll A$, then the structure of the raft changes, and in addition to the raft, a dispersion of the clusters of the second component particles is present. This situation is illustrated in Figure 4 (right panel).

We should stress the significant difference between the structure of the one- and two-component systems in the case of fixed $N_{1},N_{2}\ll A$. In the one-component case, the cluster fluid is present at temperatures down to T=0. Addition of the second component leads to assembly of the clusters into a single ’raft’ with a crystalline structure and the system becomes much more ordered.

#### 4.1.2. The Case of the Open System—Calculations

In the one-component system, the clusters or stripes are formed when the repulsion is sufficiently strong, which is the case for J>1/2. The vacuum coexists with the hexagonal arrangement of large or small clusters (see Figure 3) for 1/2<J<13/12 or J>13/12, respectively. For J>7/4, only the small clusters are formed in the ground state, because the repulsion between particles inside the same cluster made of 7 particles is too strong [19].

**Figure 5:**
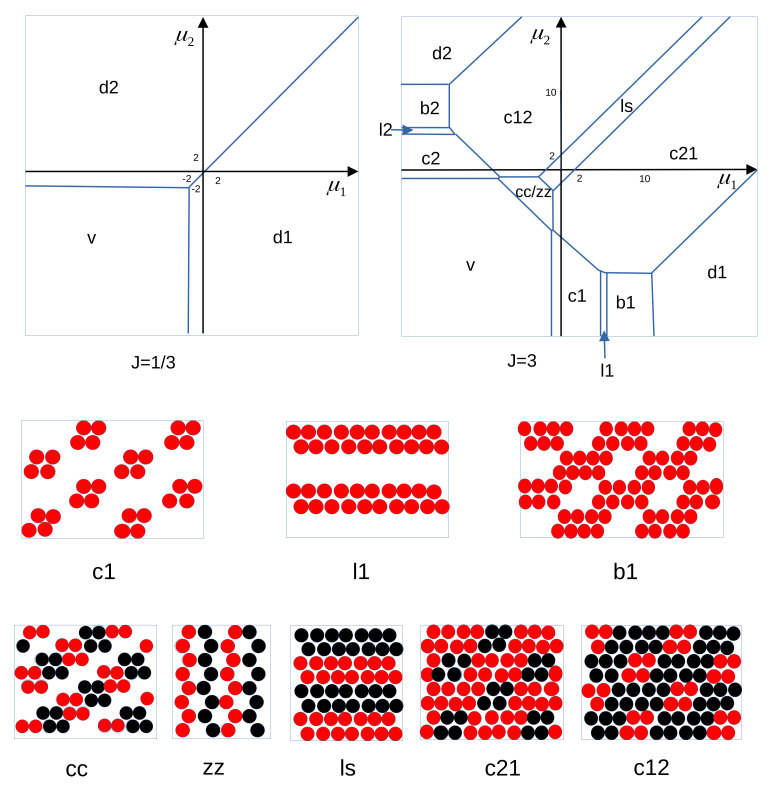
Ground state of the model for the binary mixture with the interactions given in (1) and (2). The chemical potential $\mu_{i}$ of the species *i* is in units of the nearest-neighbor interaction $J_{1}$. We compared the GS for $J=J_{2}/J_{1}=1/3$ and J=3. The periodic structures are shown below the ground states. c2, l2, b2 are the same as c1, l1, b1 with the particles of the second species replacing the particles of the first one. d1 and d2 denote the dense phases with all lattice cells occupied by the first and the second component, respectively, and v denotes the vacuum. cc/zz denotes the stability region of the coexisting cc and zz phases. Red and black circles represent the first- and the second-component particles, respectively, and empty cells are not shown.

In the two-component system, the vacuum is stable for J<1/2 when $\mu_{1}^{*}<-3+3J$ and $\mu_{2}^{*}<-3+3J$, and the phases with all lattice cells occupied by the first or the second component coexist for $\mu_{1}^{*}=\mu_{2}^{*}$ and $\mu_{i}^{*}>-3+3J$ (Figure 5). For weak third-neighbor interaction, the model has the same GS as the Blume–Emery–Griffith spin-1 model [45]. To study the self-assembly into ordered patterns, we focus on the case of J>7/4 corresponding to periodic patterns formed by small clusters [19]. Finding the lowest value of $h^{*}$ (see (4)) is a rather trivial, but tedious calculation for many different phases that may occur. We calculated $h^{*}$ for many possible patterns, and by direct comparison of the values of $h^{*}$ for all of them, we found that for J>7/4 the following phases can occur at $T^{*}=0$ (for periodic structures see Figure 5, bottom rows):

1. vacuum, v (dilute gas for $T^{*}>0$);
2. all cells occupied by the first, d1, or by the second, d2, component (disordered liquid rich in the first or the second component, respectively for $T^{*}>0$);
3. hexagonal arrangement of one-component clusters, c1, c2;
4. one-component stripes, l1, l2;
5. one-component dense phase with hexagonally ordered bubbles, b1, b2;
6. chains of alternating clusters of the two types separated by empty layers, cc;
7. alternating zig-zag chains of the first and the second component, zz;
8. alternating adjacent bilayers of the first and the second component, ls;
9. hexagonal lattice of clusters of one-component particles in the dense liquid formed by the particles of the other type, c12, c21.

For the one-component structures, $h^{*}$ was computed in ref. [19], and for the phases containing both types of the particles that are stable for J>7/4, $h^{*}$ takes the form:

(8) $$h^{*}=\left\{\begin{array}{c} -[1+3J+\mu_{1}^{*}+\mu_{2}^{*}]/3\;\;\mathrm{for}\mathrm{cc}\mathrm{and}\mathrm{zz}\;\\ -[4+4J+2\left(\mu_{1}^{*}+\mu_{2}^{*}\right)]/4\;\;\;\mathrm{for}\mathrm{ls}\;\\ -[8+12J+4\mu_{1}^{*}+8\mu_{2}^{*}]/12\;\;\mathrm{for}c12,\; \end{array}\right.$$

The first line in (8) shows that the GS is degenerated, and the phases cc and zz can coexist for their whole stability range. We do not give the many formulas for the phase coexistence lines. These lines are shown for J=1/3,3 in Figure 5, and the triple points for increasing $\mu_{1}^{*}$ are:

1. d2-b2-c12: $\mu_{1}^{*}=9/4-6J,\mu_{2}^{*}=-19/4+6J$;
2. b2-c12-l2: $\mu_{1}^{*}=9/4-6J,\mu_{2}^{*}=-5/2+3J$;
3. c12-l2-c2: $\mu_{1}^{*}=11/4-6J,\mu_{2}^{*}=-7/2+3J$;
4. c2-c12-cc: $\mu_{1}^{*}=1/4-3J,\mu_{2}^{*}=-1$;
5. c2-cc-v: $\mu_{1}^{*}=1/4-3J,\mu_{2}^{*}=-5/4$;
6. c12-cc-ls: $\mu_{1}^{*}=-3,\mu_{2}^{*}=-1$.

The remaining triple points are obtained by the replacement of the first component by the second one and vice versa.

It is worth noting that the cc-ls and cc-v coexistence lines are given by $\mu_{1}^{*}+\mu_{2}^{*}=-4$ and $\mu_{1}^{*}+\mu_{2}^{*}=-1-3J$, respectively. While the first coexistence line is independent of *J*, the second one moves towards larger $\mu_{1}^{*}+\mu_{2}^{*}$ when *J* decreases, leading to a decreasing range of stability of the cc and zz phases for decreasing *J*. The coexistence line between the c12 and c2 phases, $\mu_{1}^{*}+\mu_{2}^{*}=-3/4-3J$, changes in the same way when *J* decreases, and the two triple-points, c2-c12-cc and c2-cc-v, stay at the same distance. As a result, no coexistence between the c12 and v phases can take place for J>7/4, and the topology of the GS stays the same as in Figure 5. The distance between the triple points c2-c12-cc and c2-cc-v is very short, $\Delta \mu_{2}^{*}=1/4$, however, and can hardly be seen in Figure 5.

The GS (Figure 5) seems to be at odds with the structure of the rafts obtained in the MC simulations in the canonical ensemble. The structure of large rafts should be the same as the structure of one of the phases coexisting with vacuum, but it is different. However, in the phases with the patterns shown in Figure 4, we have $h^{*}\left(dim\right)=h^{*}\left(cc\right)/2$ and $h^{*}\left(tri\right)=h^{*}\left(cc\right)/3$, where ’dim’ and ’tri’ refer to the patterns made by dimers and triangles, respectively (Figure 4). Hence, at the v-cc/zz phase coexistence, where $h^{*}\left(cc\right)=h^{*}\left(zz\right)=h^{*}\left(v\right)=0$, the two patterns made by dimers or triangles are also stable. The stability region of these two structures is limited to the single line $\mu_{1}^{*}+\mu_{2}^{*}=-1-3J$ in the $\left(\mu_{1}^{*},\mu_{2}^{*}\right)$ diagram, however. When $h^{*}=0$ for several phases, the structure of a large raft is determined by the line tension, and it takes the minimum for the dimers and triangles for J=3 and J=2, respectively. More detailed analysis of the structure of the interface and line tensions between different phases will be performed in future studies.

#### 4.1.3. The Case of the Open System—MC Simulations

Applying the simple simulation strategy described in Section 3.1, we obtained all the structural patterns predicted by the GS calculations in different regions of the phase diagram, which now in the case of the two-component mixture is expressed in terms of the chemical potentials $\mu_{1}^{*},\mu_{2}^{*}$.

**Figure 6:**
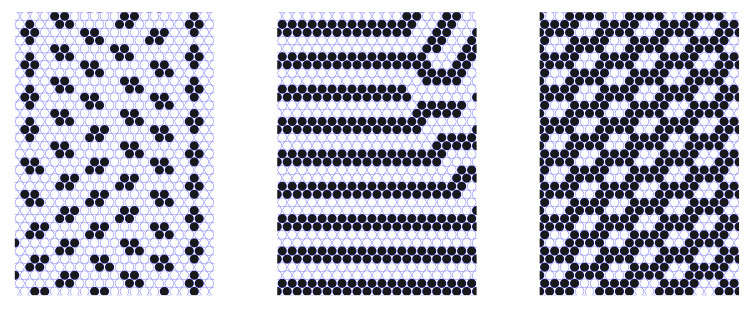
Representative configuration for the lattice model with J=3 and $\mu_{1}^{*}=-20.0$. (**Left**, **central,** and **right**) panels correspond to $\mu_{2}^{*}=0.0$, $\mu_{2}^{*}=6.0$, and $\mu_{2}^{*}=10.0$, respectively. Black spheres represent type-2 particles, and open circles represent empty cells. A part of the simulation box is shown.

In the first step we set $\mu_{1}^{*}=-20$, and increase the chemical potential of the second component. For this rather low value of the chemical potential, no type-1 particles are observed and the lattice becomes increasingly covered by type-2 particles. The progression c2 → l2 → b2 is obtained, in agreement with the GS (Figure 6).

**Figure 7:**
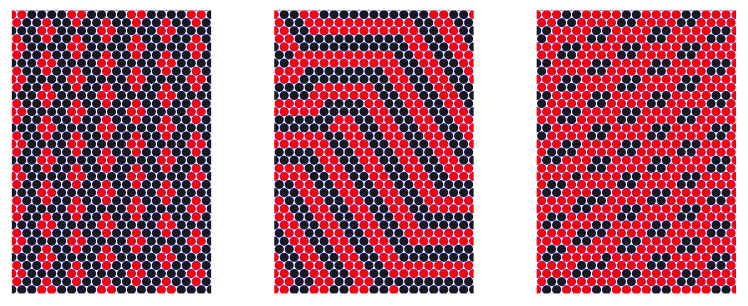
Representative configuration for the lattice model with J=3 and $\mu_{2}^{*}=10.0$. (**Left**, **central,** and **right**) panels correspond to $\mu_{1}^{*}=0.0$, $\mu_{1}^{*}=10.0$, and $\mu_{1}^{*}=20.0$, respectively. Red and black spheres represent type-1 and type-2 particles. A part of the simulation box is shown.

We also performed a scan at a moderate value of the chemical potential for one of the species, namely $\mu_{2}^{*}=10.0$, as shown in Figure 7. As the value of $\mu_{1}^{*}$ is increased, the predicted c12 → ls → c21 progression of phases is clearly seen. The lamellar structure at $\mu_{1}^{*}=\mu_{2}^{*}=10.0$ displays several defects (disclinations), which originate at the annealing process. To remove these defects requires the use of more involved simulation techniques and this is out of the scope of the present work.

**Figure 8:**
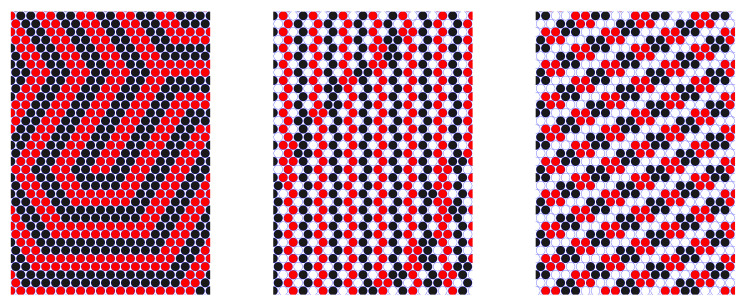
Representative configuration for the lattice model with J=3 and $\mu_{1}^{*}=\mu_{2}^{*}$. (**Left**, **central,** and **right**) panels correspond to $\mu_{1}^{*}=\mu_{2}^{*}=0.0$, $\mu_{1}^{*}=\mu_{2}^{*}=-3.2$, and $\mu_{1}^{*}=\mu_{2}^{*}=-3.8$, respectively. Red and black spheres represent type-1 and type-2 particles, and~open circles represent empty cells. A part of the simulation box is shown.

In Figure 8 we present the case of identical chemical potentials for both types of particles ($\mu_{1}^{*}=\mu_{2}^{*}\equiv \mu^{*}$) in order to describe the ls → cc/zz → v phase progression. Starting with $\mu^{*}=0.0$, a lamellar structure build-up of multiple domains with different orientations is obtained, which is consistent with the ls phase. Note that for this rather low value of the chemical potential, the lattice still remains fully covered, i.e., there are no empty sites. On lowering $\mu^{*}$, we move through a structure that combines different types of long-range order for both components, with most of the area covered by the zz structure for $\mu^{*}=-3.2$, which is inside the coexistence region of the cc and zz phases on the GS ($-1-3J<\mu_{1}^{*}+\mu_{2}^{*}<-4$). Inclusions of chains of alternating clusters with the structural motifs of the coexisting cc and metastable ls phases can result from T>0 and degeneracy of the GS (Figure 8, central panel). Finally, as we reach the value $\mu^{*}=-3.8$, the cc phase is obtained (Figure 8, right panel).

### 4.2. The Continuous Model

**Figure 9:**
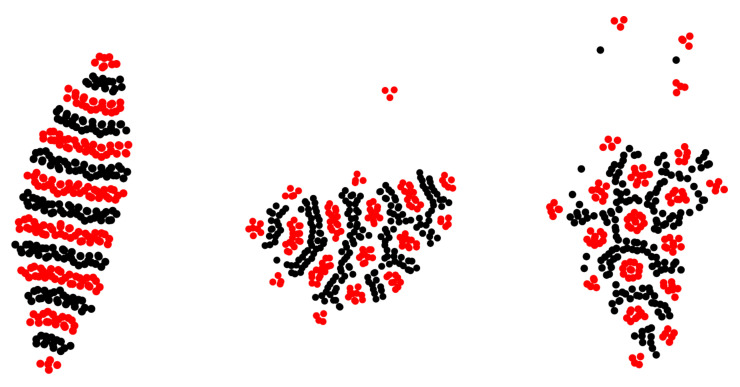
Representative configurations in the layer self-assembled between repulsive walls in the continuous model (3) for $N_{1}=N_{2}=200$, $L_{x}=L_{y}=150$, and $L_{z}=4$. A projection on the (x,y) plane is shown for a part of the simulated system. The red and black circles represent the first and the second component. (**Left**) raft: q=1 and $\bar{T}=0.12$. (**Central**) raft: q=1/2 and $\bar{T}=0.06$. (**Right**) raft: q=1/2 and $\bar{T}=0.08$.

The patterns obtained in our MD simulations for the particles interacting with the potentials (3) and located between two repulsive walls are shown in Figure 9 for $N_{1}=N_{2}=200$. We compared the structures self-assembled inside the slit with the horizontal sizes $L_{x}=L_{y}=150$ and the width $L_{z}=4$, for symmetric (q=1) and asymmetric (q=1/2) interactions. In both cases, the majority of the particles aggregate into a large cluster (a ‘raft’) in the center of the slit. The thickness of the raft in the *z*-direction is between 1a and 2a.

For q=1 (Figure 9, left panel), the raft has the shape of a symmetrical spindle, and consists of alternating parallel stripes perpendicular to the symmetry axis of the raft and separated by thin empty layers. The shape is determined by the line tension. It is larger in the direction parallel to the stripes, due to the missing attraction between stripes of different components.

When q=1/2, then the optimal distribution of the particles is no longer symmetrical. The stronger repulsion between first-component particles at large distances makes it favorable to break their stripes into clusters, whereas weaker repulsion between the particles of the second component allows for making a branched network surrounding the particles of the first kind. Such a structure, however, would be associated with a smaller number of the particles of the first component if the thickness of the stripes or clusters should remain ∼2a. For $N_{1}=N_{2}$, there is a frustration between the numbers of the particles and the optimal structure. For $\bar{T}=0.06$, the raft consists of two parts: in the first one, clusters of strongly interacting particles are surrounded by the particles of the other component; in the other one, alternating stripes are present. For $\bar{T}=0.08$, the hexagonal arrangement of the clusters of the first-component particles is surrounded by the network of the second-component particles. The size of the clusters is larger, and the thickness of a part of the layers of the second component is thinner than 2a to accommodate nearly equal numbers of the particles of the two components. In the gas surrounding the raft, the small clusters are formed only by the strongly interacting particles. The results for the internal structure of the rafts presented in Figure 9 have been confirmed by simulations on four-times larger systems ($N_{1}=N_{2}=800,L_{x}=L_{y}=300,L_{z}=4.0$).

The structure in the monolayers belonging to the film adsorbed on a surface was studied in a large box, with $L_{x}=L_{y}=200$ and $L_{z}=800$. The adsorbed film can be considered a subsystem in equilibrium with the reservoir of particles in the rest of the box.

**Figure 10:**
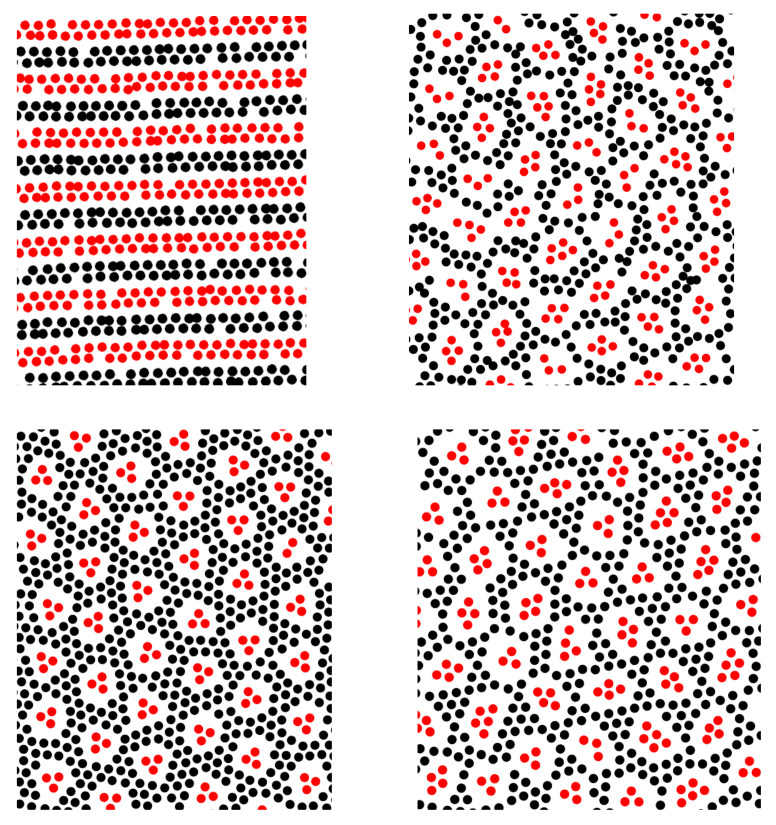
Representative configurations for the continuous model. A projection on the (x,y) plane of a layer of particles of thickness ∼1a is shown for a part of the simulated system. The red and black circles represent the first and the second component. (**Left**) panel in the (**top**) row: the slab inside a monocrystal for q=1 ($N_{2}=N_{1}$) at $\bar{T}=0.14$. (**Right**) panel in the (**top**) row: particles at the distance 1.7<z<2.7 from the wall attracting the first component for q=1/2 ($N_{2}=2N_{1}$ = 21,334) and $\bar{T}=0.03$. (**Bottom**) row: the monolayer adsorbed at the wall attracting both components. (**Left**) panel: q=1/3, $N_{2}=3N_{1}$ = 24,000, and $\bar{T}=0.03$. (**Right**) panel: q=1/2, $N_{2}=2N_{1}$ = 21,334, and $\bar{T}=0.04$.

In Figure 10, we compare the structure in a slab inside the 3D crystal formed for q=1 and $N_{1}=N_{2}$ at $\bar{T}=0.14$ with the patterned monolayer of particles in the film adsorbed on a solid surface. We show a projection on the (x,y) plane of the particles in the layer of thickness 1a. In the case of the adsorbed film, we assume q=1/2 or q=1/3, and $N_{1}=N_{2}/2$ or $N_{1}=N_{2}/3$ in the whole system, respectively. For q=1/2, we compare patterns self-assembled at a surface attracting the first component, and at a surface attracting both components. In the case of the selective surface, the center of the layer of thickness 1a is at the distance z=2.2a from the wall. For the wall attracting both components, we show the first monolayer adsorbed at the surface. Note the similarity between the phase shown in the upper-left panel in Figure 10 and the ls phase in our lattice model (see Figure 5 (ls pattern), Figure 7 (central panel), Figure 8 (left panel)). The thickness of the empty layers between the particles is smaller than the particle diameter, therefore they cannot be observed on the lattice. The remaining structures shown in Figure 10 resemble strongly our c12 phase (see Figure 5 (c12 pattern), Figure 7 (left panel)). The order for T>0 is not perfect, but we can see small clusters of the first component consisting on average of 4 or 3 particles inside the bubbles formed in the liquid of the second component for q=1/2 or q=1/3. The bubbles with the clusters inside them form a hexagonal lattice with some defects.

## 5. Summary and Conclusions

We studied a binary mixture of oppositely charged particles with additional short-range attraction between like particles, and short-range repulsion between different ones. The interactions in the model are motivated by the interactions between charged inclusions in the membranes of living organisms. Our aim was to determine possible patterns that may occur in monoloayers of particles with such interactions, and to identify the main factors that govern the pattern formation. Such competing interactions are present not only between inclusions in the biological membranes but also between charged nanoparticles or quantum dots with solvent-induced short-range attraction between like particles and repulsion between different particles. Our results may concern pattern formation at solid surfaces or at fluid interfaces that can be of practical importance.

We proposed a very simple triangular lattice model with full symmetry between the two components, and with the interactions having the key properties of the considered mixtures, Equations (1) and (2). We focused on the patterns minimizing the energy for a fixed number of particles, or minimizing the grand potential for fixed chemical potentials and T→0. We compared the predictions of our 2D lattice model with the results of the MD simulations of a binary mixture with the continuous interactions (3), and with the same or different interactions between particles of the first and the second component, q=1 or q=1/2,1/3.

In the continuous model, we examined the structure of a ’raft’ self-assembled between parallel repulsive walls separated by a short distance, as well as in a monolayer of particles extracted from a self-assembled monocrystal, and in a monolayer parallel to a surface adsorbing particles either of the first component or of both components.

We found that despite the restrictions due to the lattice structure, our model reproduces the structures observed in the continuous model quite well for a high density of the particles. Moreover, a higher strength and shorter range of the repulsive part of the interactions in the lattice case does not have a strong influence on the results for high densities. In the continuous 3D model, however, the gas phase coexists with the dense phase with alternating bilayers of the two components, and by examining the structure for $N_{1}=N_{2}$, we could see neither the chains of alternating clusters nor the thin zig-zag chains of like particles separated by empty regions that are predicted by the lattice model. The structure of the rafts that appear in the lattice model was not seen in the continuous model either. Interestingly, the structure of these self-assembled objects is quite different for different strengths of the third-neighbor interaction on the lattice. It is not clear yet if these more complex patterns are artifacts of the lattice structure, or result from the difference in the interaction ranges in our two models. This question requires further investigation.

The short-range repulsion and long-range attraction between the particles of different species resembles interactions between core-shell particles adsorbed at an interface [46,47,48,49]. The patterns and phase coexistence obtained in triangular lattice models for such particles with different ranges and shapes of the attractive and repulsive parts of the interaction were significantly different. Interestingly, the patterns predicted by the lattice models with shorter and longer ranges of the interactions agreed quite well with experiments for particles with thinner and thicker shells, respectively [48,49]. A similar variety of patterns may be expected for our case. It is thus interesting to investigate how the range and strength of the interactions influences the low-density part of the phase diagram and the structure of the rafts.

We conclude that for high densities, the patterns in mixtures of charged particles with short-range attraction between like particles and repulsion between different particles depend mainly on the ratio $N_{1}/N_{2}$ inside the monolayer, and not on the method that this ratio achieved (chemical potential difference, selectivity of the adsorbing surface or asymmetry in interactions). In addition, neither the underlying lattice nor the range of the interactions influence the structure of the ordered phases. For low densities or chemical potentials, however, the detailed form of the interactions plays a very important role, and the monocrystals (’rafts’) of the phases coexisting with dilute gas have different structures and shapes in the lattice and continuous models. In addition, different strengths of the repulsion between like particles and attraction between different ones lead to quite different patterns in the self-assembled aggregates. The dependence of the structure of the self-assembled rafts on the shape of the interactions requires further study going beyond the scope of this work.

Another open question is the effect of the structure of the underlying lattice on the self-assembled patterns. We considered a triangular lattice, because it allows for close packing of spherical particles. For particles with different shapes, different lattices may be more appropriate. In particular, square or rectangular lattices may be better suited for nanoparticles that have rectangular shapes. For a one-component SALR model, chess-board and striped patterns were found in ref. [50] on a square lattice. The patterns formed in two-component systems on different lattices have not yet been investigated. We are convinced that in binary mixtures with competing interactions, a rich variety of ordered patterns with different symmetries and structural motifs are yet to be discovered.

## Data Availability

Not applicable.
